# First report of *Aedes albopictus* in Saint Barthélemy (French West Indies) confirmed by morphological, molecular and MALDI-TOF mass spectrometry approaches

**DOI:** 10.1186/s13071-025-06916-7

**Published:** 2025-07-04

**Authors:** Cédric Ramdini, Elodie Calvez, Olivier Houy, Christophe Gréaux, Christelle Dollin, Nicolas Pocquet, Lionel Almeras, Fabrice Sonor, Gérald Déliscar-Jourdan, Anubis Vega-Rúa

**Affiliations:** 1 Agence Régionale de Santé Guadeloupe, Saint-Martin, Saint Barthélemy, Guadeloupe France; 2 Institut Pasteur de Guadeloupe, Guadeloupe, France; 3 Cellule de Lutte Antivectorielle des Îles du nord, Saint Barthélemy, France; 4 Institut Pasteur de Nouvelle-Calédonie, Nouméa, New Caledonia; 5 Département Microbiologie et Maladies Infectieuses, Unité Parasitologie et Entomologie, Institut de Recherche Biomédicale Des Armées, Marseille, France; 6 Aix-Marseille Université, SSA, AP-HM, RITMES, Marseille, France; 7 Institut hospitalo-universitaire en maladies infectieuses de Marseille (IHU Méditerranée Infection), Marseille, France; 8 Agence Régionale de Santé Martinique, Martinique, France; 9 Cellule de Lutte Antivectorielle des Îles du nord, Saint Martin, France

**Keywords:** *Aedes albopictus*, Mosquito, Invasive species, Saint Barthélemy

## Abstract

*Aedes albopictus* is a mosquito vector of arboviruses that is native to southeast Asia. However, this invasive species has spread worldwide. It arrived in the Caribbean in 1993, but had never been recorded in the French Territories of the Americas. We report here the first detection of *Aedes albopictus* in Saint Barthélemy, Guadeloupe confirmed by morphological criteria, matrix-assisted laser desorption ionization—time-of-flight mass spectrometry (MALDI-TOF MS), and cytochrome* c* oxidase 1 (*cox1*) gene barcoding. The presence of this invasive mosquito species in Saint Barthélemy, an island with daily aerial or maritime connections to the French Departments of the Americas, raises concerns about the risk of its introduction into these territories, as well as into other Caribbean countries. It also emphasizes the urgent need to locally reinforce vector surveillance and control measures to prevent the further spread of this mosquito vector.

**Graphical abstract:**

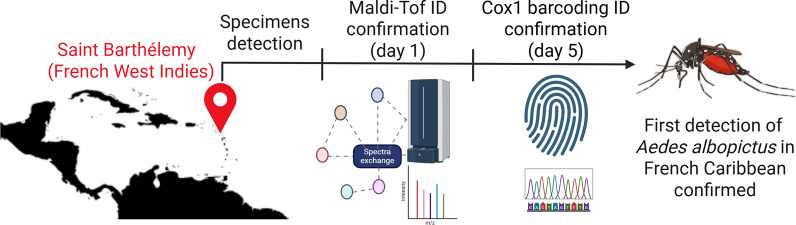

The French Territories of the Americas are regions frequently impacted by arthropod-borne viruses transmitted by *Aedes aegypti* mosquitoes, such as dengue, chikungunya, and Zika viruses [1]. However, the presence of *Aedes albopictus*, another important mosquito vector for arboviruses [2], had never been reported in any of these territories, despite the impressive spread of this mosquito in mainland France since 2004 [3]. In the Caribbean, *Ae. albopictus* was first reported in the Dominican Republic in 1993 [4].

On 30 September 2024, two vector control agents collected mosquito larvae from a floor siphon (17°54′20″N, 62°49′28″W) near the Lorient post office on the island of Saint Barthélemy, as part of their routine entomological surveillance activities that include weekly larval prospections and ovitrapping. The larvae were brought to the Vector Control Agency office (Agence Régionale de Santé [ARS]) in Saint Barthélemy and reared until adulthood. After emergence, the agents noted the presence of adult mosquitoes that did not match morphological criteria for *Ae. aegypti* and photographed the mosquitoes. Entomologists from ARS Guadeloupe, ARS Martinique and from local research institutions examined the photographs and confirmed the agents' suspicion that the mosquitoes were *Ae. albopictus*.

Two weeks later, an exploration mission was organized, during which agents from ARS Guadeloupe, ARS Saint Barthélémy, and the Institut Pasteur of Guadeloupe (IPG) traveled to Saint Barthélémy to confirm the species’ identity and to inform about its distribution area on the island. Larval prospections conducted from 14 to 16 October identified five breeding sites within a 400-m radius of the initial detection site, all containing mosquitoes morphologically identified as *Ae. albopictus* (Table 1).

**Table 1:** *Aedes albopictus* breeding sites identified in Saint Barthélemy (French West Indies) between 14 and 16 October 2024

Breeding site	Coordinates	Species identified^a^
Floor siphon (post office)	17°54′20″N, 62°49′28″W	*Aedes aegypti; Aedes albopictus*
Tire	17°54′16″N, 62°49′27″W	*Ae. aegypti* (6); *Ae. albopictus* (2; males)
Watering can	17°54′15″N, 62°49′32″W	*Ae. albopictus*
Flowerpot dish	17°54′21″N, 62°49′29″W	*Ae. aegypti* (10); *Ae. albopictus* (1; female)
Small container	17°54′24″N, 62°49′15″W	*Ae. albopictus*

^a^The number of specimens (when available) is given in parentheses

Morphological criteria described by Darsie [5] were initially used during the missions to identify *Ae. albopictus* at both the larval and adult stages (Fig. 1a–c). However, since *Ae. albopictus* shares morphological similarities with other members of the Scutellaris group, further identification steps were performed on two collected specimens using matrix-assisted laser desorption ionization—time-of-flight mass spectrometry (MALDI-TOF MS) and cytochrome* c* oxidase 1 (*cox1*) gene barcoding to confirm the species. Briefly, the two specimens were individually dissected to separate the head and thorax from the rest of the body, and then homogenized 3 × 1 min at 30 Hz in a tissue lyzer (Retsch GmbH, Haan Germany). Legs and/or thoraxes were separately treated for identification by MALDI-TOF MS as previously described [6]. Protein mass profiles were acquired using a Maldi Biotyper Sirius Mass Spectrometer (Bruker Daltonics, Bremen, Germany), operating in linear positive-ion mode, with detection at a laser frequency of 50 Hz within a mass range of 2–20 kDa. MALDI-TOF spectra databases shared between entomologists from the Institut Pasteur of New Caledonia and the IHU Méditerranée enabled almost instantaneously confirmation of the samples as *Ae. albopictus* species, with the relevant score for the two specimens (log-score identification value = 2.05, matching spectra of *Ae. albopictus* from Cameroon [using mosquito legs from specimen 1] and from Marseille [using mosquito thorax from specimen 2]). To evaluate spectral similarity between specimen 1, which was collected in Saint-Barthélemy, and those from other *Ae. albopictus*, we generated a clustering analysis via the MSP (main spectra library) dendrogram function in MALDI-Biotyper v3.0 software. (Bruker Daltonics) using MS spectra from *Ae. albopictus* and five other *Aedes* species from the Scutellaris group (*Aedes scutellaris*, *Aedes pseudoscutellaris, Aedes futunae, Aedes malayensis* and *Aedes polynesiensis*). The resulting MSP dendrogram showed that the spectrum of the Saint-Barthélemy specimen clustered together with those of other *Ae. albopictus*, ruling out the possibility of other species from the Scutellaris group (Fig. 1d). These results highlighted the high reproducibility and the specificity of protein profiles among *Aedes* species, allowing a rapid and relevant identification of the specimen.

**Fig. 1:**
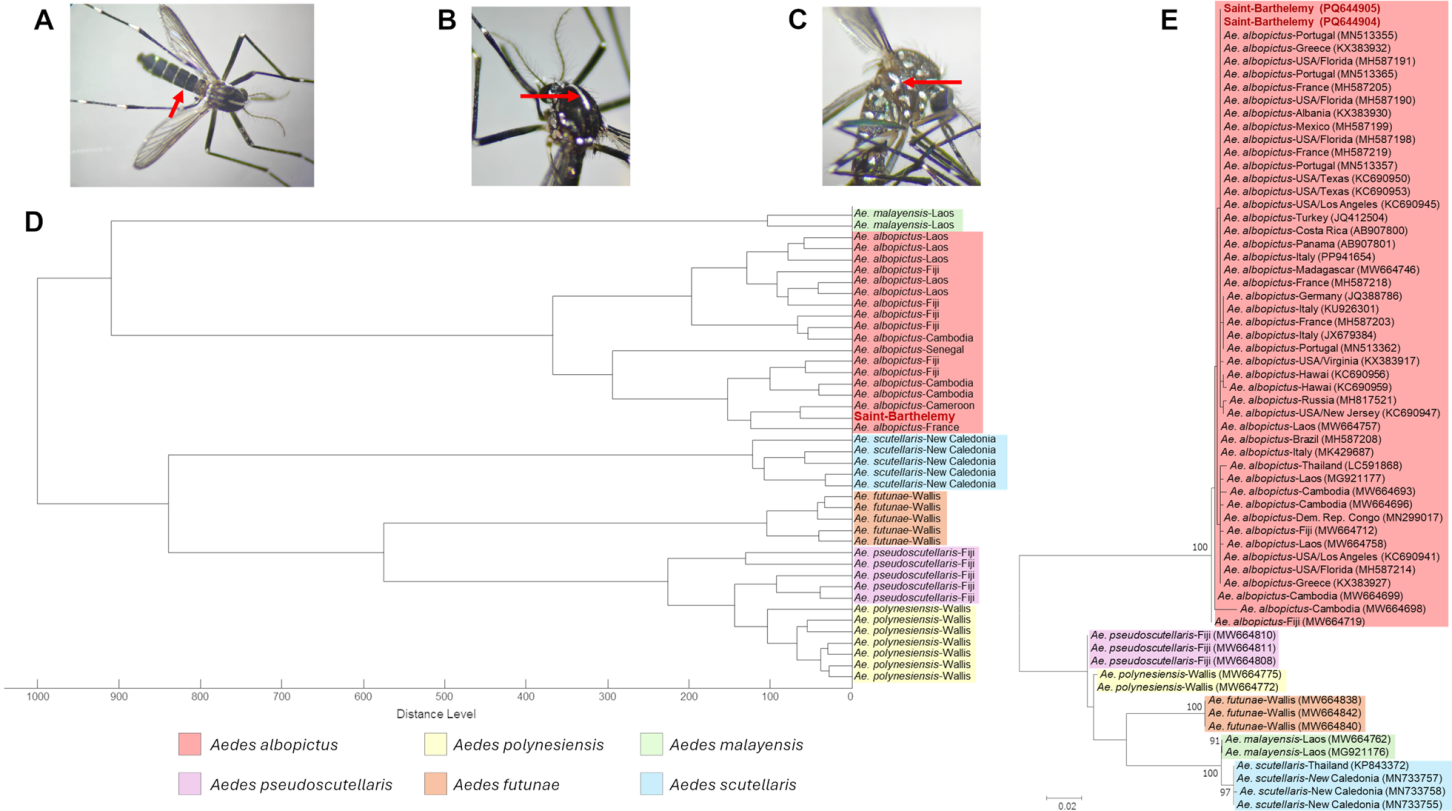
Identification of adult *Aedes albopictus* specimens found in Saint Barthélémy (French West Indies) according to morphological criteria (**A**, **B**, **C**), MALDI-TOF MS analysis (**D**) and *cox1* gene barcoding (**E**). **A** Abdomen view: abdominal terga with complete basal white bands (red arrow), **B** thorax view: scutum with a medial-longitudinal white stripe (red arrow), **C** thorax view: mesepimeron with non-separated white scales, forming a V-shaped white spot (red arrow). **D** Main spectrum profile (MSP) dendrogram of *Aedes* Scutellaris Group. Spectrum from the mosquito specimen collected in Saint Barthélémy was obtained with a Maldi Biotyper Sirius mass spectrometer (Bruker Daltonics), and the dendrogram was generated by MALDI Biotyper Compas Explorer software. Distance units correspond to the relative similarity between spectra of *Ae. albopictus* from Saint Barthélémy and those from IHU Méditérranée and Institut Pasteur New Caledonia databases. **E** Molecular phylogenetic tree generated by the maximum likelihood method. The percentage bootstrap values shown at the nodes were calculated with 1000 replicates, and only bootstrap values > 70 are shown. Scale bar indicates nucleotide substitutions per site. *Aedes albopictus* sequences or spectra from Saint Barthelemy specimens are indicated in red and bold. *cox1*, Cytochrome* c* oxidase 1 gene; IHU, Institut hospitalo-universitaire; MALDI-TOF MS, matrix-assisted laser desorption ionization—time-of-flight mass spectrometry. Mosquito pictures were taken by Fabrice Sonor

We subsequently used the remaining body parts from the two specimens to conduct *cox1* barcoding. Total DNA was extracted using the cetyltrimethylammonium bromide technique as previously described [7] and sequenced by Eurofins Genomics Europe (Sanger sequencing) using the primers HCO2198 and LCO1490 [6]. Sequences were analyzed, and multiple sequence alignment (Clustal W) was conducted using BioEdit version 7.0.5.3 software (BioEdit, Manchester, UK) [8, 9]. A molecular phylogenetic tree was generated by the maximum likelihood method based on the Timura 3-parameter model (best-fit nucleotide substitution pattern determined according to the corrected Akaike Information Criterion) using MEGA (http://www.megasoftware.net) with a bootstrap of 1000 replications [10]. Barcoding results indicated that the sequences from the mosquitoes collected in Saint Barthélemy (GenBank accession no. PQ644904 and PQ644905) shared 100% homology with sequences from *Ae. albopictus* collected in Europe and the Americas (Fig. 1e).

In the following weeks, weekly entomological surveillance continued, and the number of *Ae. albopictus*-positive breeding sites on Lorient increased to 18 in a 665-m^2^ area. The species’ presence was also detected in five breeding sites within a 150-m^2^ area in Saint Jean (17°53′50″N, 62°50′14″W). The discovery of this second hotspot, located 1.6 km from Lorient and near the airport, suggests a broader distribution of the species on the island. The presence of this invasive mosquito species in Saint Barthélémy, an island with daily aerial or maritime connections to the French Departments of the Americas [11], raises concerns about the risk of its introduction into these territories, as well as into other Caribbean countries. After the first detection of *Ae. albopictus* in the Dominican Republic in 1993 [4], the species has been reported in an increasing number of Caribbean territories, including Cuba in 1995 [12], Cayman Islands in 1997 [13, 14], Trinidad in 2002 [15], Haiti in 2010 [16], and Jamaica in 2018 [17] (Fig. 2).

**Fig. 2:**
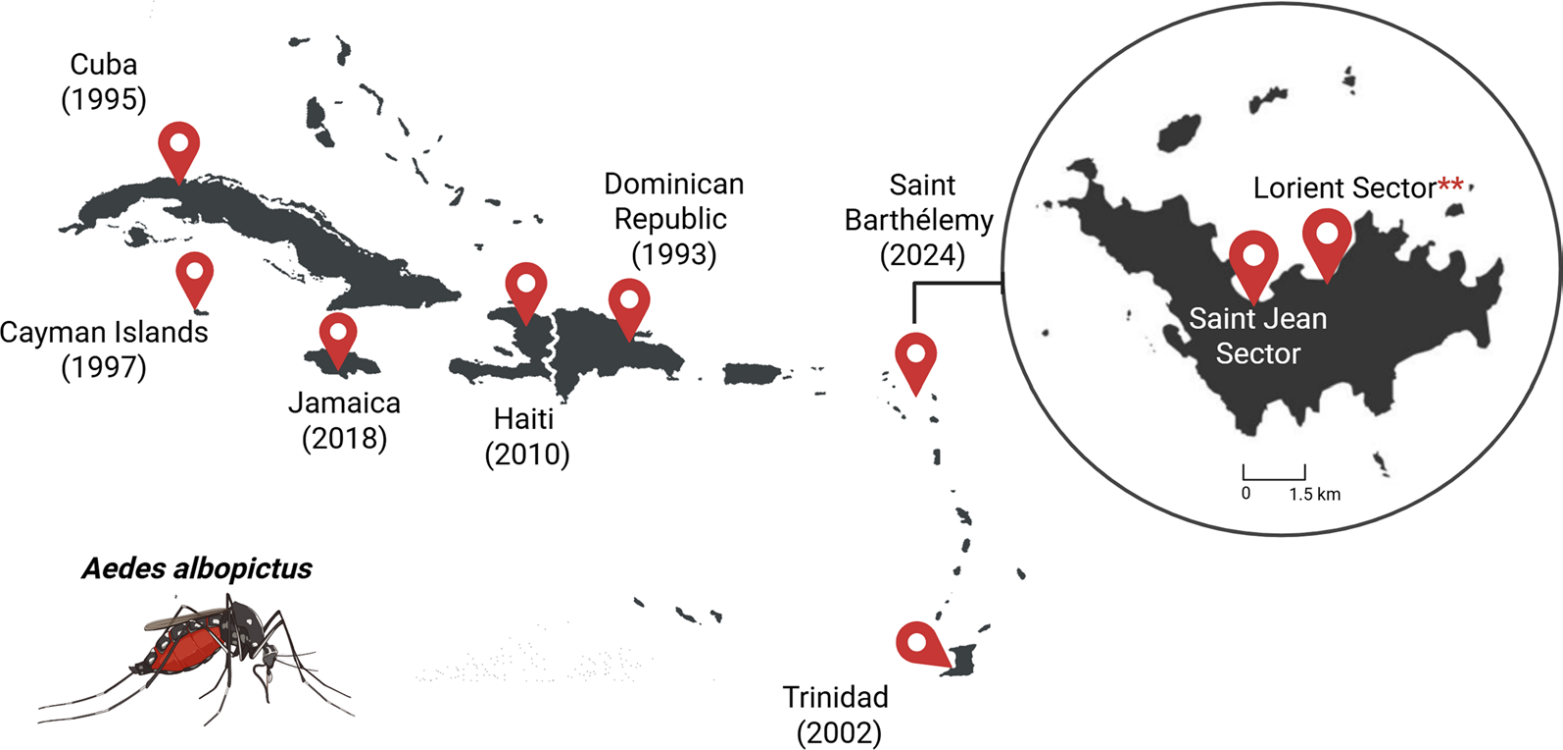
*Aedes albopictus* distribution in the Caribbean. Countries or territories reporting the species have been marked with red location pins. Date of first detection is given in parentheses. Asterisks indicate the first detection in Saint Barthélemy. *cox1*, Cytochrome* c* oxidase 1 gene; IHU, Institut hospitalo-universitaire; MALDI-TOF MS, matrix-assisted laser desorption ionization—time-of-flight mass spectrometry. Created with BioRender.com

It is noteworthy that in Cuba, from 1995 to 1999, *Ae. albopictus* was mainly distributed in peripheral municipalities with abundant vegetation rather that in more urbanized areas (i.e. city center). However, an increase in the species’ distribution in more urbanized areas, in association with *Ae. aegypti*, was observed from 2010 to 2018, highlighting the competitiveness of *Ae. albopictus* in the Caribbean context and an invasion process that can take several years [18]. In Saint Barthélemy, a 25-km^2^ island, *Ae. albopictus* is already present in artificial breeding sites from urban settings. In addition, half of these breeding sites contained the species in association with *Ae. aegypti* individuals (Table 1). In this context, the ecological plasticity of *Ae. albopictus* [19, 20] will likely facilitate its rapid invasion of Saint Barthélémy, emphasizing the urgent need to locally reinforce vector surveillance and control measures to prevent the further spread of this mosquito.

This work also highlights the efficiency of MALDI-TOF MS for rapidly identifying mosquito specimens in a real-life surveillance situation. The value of this approach for entomological surveillance has been repeatedly shown [21, 22]. Although MALDI-TOF MS-based identification requires reference spectra databases to identify specimens, as well as standardized protocols for spectrum acquisition [23], the exchange of databases between regions and institutes with access to different mosquito species represents a key development perspective. This is particularly relevant in our increasingly globalized world, where the potential for transcontinental movements of viruses and vectors continues to grow.

## Data Availability

The datasets generated and/or analyzed during the current study are available in Genbank database (http://www.ncbi.nlm.nih.gov/).
